# An Evaluation of Large Language Models for Supplementing a Food Extrusion Dataset

**DOI:** 10.3390/foods14081355

**Published:** 2025-04-15

**Authors:** Necva Bölücü, Jordan Pennells, Huichen Yang, Maciej Rybinski, Stephen Wan

**Affiliations:** 1 CSIRO Data61, Sydney, NSW 2122, Australia; necva.bolucu@csiro.au (N.B.); huichen.yang@csiro.au (H.Y.); maciek.rybinski@csiro.au (M.R.); 2 CSIRO Agriculture and Food, Food Innovation Centre, Werribee, VIC 3030, Australia; jordan.pennells@csiro.au

**Keywords:** natural language processing, extrusion, food processing, large language model

## Abstract

Food extrusion is a widely used processing technique that transforms raw ingredients into structured food products—foods with well-defined textures, shapes, and functionalities—through mechanical shear and thermal energy. Despite its broad industrial application, the absence of a standardised, structured dataset capturing extrusion research parameters has hindered research synthesis, product development, and process optimisation. To address this gap, we introduce a manually curated food extrusion literature dataset capturing publication details, product types, process parameters, formulation data, experimental variables, characterisation metrics, and study-level insights. However, while manually curated datasets are typically of high quality, their scope is limited by time and resource constraints. We propose a method to supplement the dataset using large language models (LLMs) and evaluate the accuracy of LLMs in extracting structured food extrusion data from the scientific literature. Our findings demonstrate that LLMs can effectively extract structured information. However, some challenges, such as hallucination and missing contextual details, remain, suggesting that human effort can be spent on validating the resulting data. This still represents significant time savings as validation is a less time-consuming task than data extraction. We argue that LLMs thus represent a viable tool in providing supplementary datasets, and we propose a method to leverage existing human efforts in dataset creation to improve data quality.

## 1. Introduction

Food manufacturing employs a diverse range of processing techniques—including drying, frying, roasting, baking, extrusion, fermentation, canning, and sterilisation—to transform raw agricultural products into consumable goods with enhanced nutrition, safety, flavour, and shelf life. These methods ensure year-round access to nutritious and palatable food options for consumers while addressing global challenges of food security and sustainability. As a result, the food processing sector plays a significant role in the global economy, supporting increased food supply to meet population growth and adapting to evolving consumer preferences.

*Extrusion* is a widely used process in the food industry, combining multiple operations such as mixing, cooking, and shaping to convert raw ingredients into their final product form [1,2]. Researchers in this field document key aspects of extrusion processing, including formulation details (i.e., ingredients, moisture content), processing conditions (i.e., throughput, screw speed, barrel temperature), equipment setups (i.e., screw configuration, die dimensions), and the resulting extruded product attributes (i.e., colour, texture, sensory properties) [3,4]. However, this information is often presented in unstructured formats and not curated across multiple studies, making it difficult to systematically extract, compare, and analyse key experimental parameters across the literature.

To address this gap, our first contribution is the release of a human-annotated food extrusion dataset of published research articles, curated by a domain expert with a background in food engineering. This dataset captures critical parameters relevant to food extrusion research, organised under a predefined schema, a structure that defines the structured format or categories into which the extracted information is organised [5,6], to ensure consistency and comparability across studies. The dataset includes key parameters related to food extrusion processes, such as extruder details, product formulations, and characterisation methods, providing a comprehensive overview of the field (see Section 2.1). Additionally, the dataset is essential for organising research insights, enabling systematic comparison of studies, and supporting meta-analysis of processing techniques. It also allows researchers and industry technologists to track trends in ingredient usage, experimental conditions, and characterisation methods, facilitating more efficient literature synthesis and identification of research gaps. This dataset resource can provide practical value for food processing enterprises by establishing baseline processing conditions to use, based on what has worked in published studies, identifying common characterisation methods for product evaluation, and highlighting product and research gaps to enhance competitive intelligence through benchmarking and refined production strategies.

In creating this dataset, however, we observed that manual data extraction will always be limited. Because significant effort is required to review hundreds of articles, summarise findings, and organise information, any such effort only considers a limited subsection of the literature due to time and labour costs. In food science, there are semantic resources such as FoodData Central (FDC) [7] (https://fdc.nal.usda.gov/ (accessed on 20 March 2025)), Food Database (FooDB) (https://foodb.ca/ (accessed on 20 March 2025)), and AGROVAC [8] (https://www.fao.org/agrovoc/ (accessed on 20 March 2025)). These resources contain structured information on food composition, nutrients, bioactive compounds, agricultural terminologies, and related scientific concepts. However, the scientific literature is constantly evolving, introducing new concepts, terminologies, and relationships that are not immediately integrated into these resources. As such, these resources are not well suited for extracting information from recent scientific articles, as they do not provide real-time updates on new extrusion research or experimental findings, which can be directly extracted from those articles. This limitation highlights the need for advanced methods to process and interpret newly published research articles.

To address this challenge, our second contribution is a proposal of a method using artificial intelligence (AI) to provide supplementary data for a manually annotated dataset, demonstrating this on the food extrusion dataset that we introduce here. Supplementary data refers to additional data generated by our proposed method to extend the existing dataset. Specifically, we focus on the recent AI development of *large language models* (LLMs), models of human language based on web-sized training data, which allow in-depth information queries on a broad range of topics and text data [9,10,11]. LLMs, such as GPT [11], Claude [12], Gemini [13], and Llama [10], have demonstrated remarkable performance across various natural language processing (NLP) tasks, particularly in *zero-shot* and *few-shot* learning settings [9,14,15,16]. Zero-shot learning allows models to handle tasks without task-specific examples, relying on pre-trained knowledge and instructions. Few-shot learning, on the other hand, uses a small set of examples to guide predictions in addition to pre-trained knowledge and instructions. This approach utilises the ability of LLMs to accept long requests (referred to as *large context windows*) for information (e.g., questions) that can include extensive contextualisation for the request, such as prototypical examples representing the kind of data to be extracted. This mechanism significantly enhances the utility of LLMs in scientific information extraction (IE), demonstrating their capability to transform unstructured data from sources like scientific articles, reports, and reviews into structured formats [17,18,19]. This approach has been explored across various fields, including materials science [20], computer science [21], agriculture [22], and chemistry [23]. The ability of LLMs to process vast amounts of unstructured text and transform it into structured formats makes them an ideal tool for providing supplementary material to human-annotated datasets. That is, LLMs represent an efficient and scalable approach to tackle the data acquisition bottleneck.

In this work, we present the manually-annotated dataset and describe our method to leverage the existing human-provided data (manually extracted details of food extrusion) in order to generate supplementary data. By using LLMs, our approach enables the models to learn from the human-annotated data and expand them, creating a more comprehensive and dynamic dataset. This method addresses the challenge of building datasets, which is often beyond the scope of manual effort alone. While human annotation is crucial for ensuring accuracy and relevance, our approach provides a scalable solution for generating data at a large scale, significantly reducing the limitations of human labour and time constraints. Our study focuses on food extrusion research, addressing the following key questions:

- **RQ1:** What are the key challenges of manually curating high-quality structured datasets for food extrusion research, and how can LLMs address these challenges?
  Manually curating high-quality structured datasets for food extrusion research presents several challenges. It is time-consuming, resource-intensive, and limited by human capacity, making it difficult to create comprehensive datasets that can keep pace with the growing volume of research. LLMs address these challenges by automating data extraction, significantly increasing the dataset size beyond what is feasible with manual effort alone. We present an approach that uses LLMs to extract information by learning from the human-annotated data (see Section 2). By automating the process, LLMs reduce the time and effort required for dataset curation, enabling the creation of dynamic, scalable datasets. The domain expert reported that automation can reduce manual effort by up to 50%, requiring only verification of the extracted information for correctness, which aligns with the outcome of Wan et al. [24].
- **RQ2:** How do LLMs perform in automating the extraction of domain-specific information from the scientific literature compared to manual human annotation?
  Our experiments showed that LLMs effectively identify key details about product information, process and formulation details, and variables and characterisation methods. By using a few-shot learning approach with domain expertise, we improved the accuracy and reliability of LLM-generated data. LLMs significantly reduce the time and effort required—potentially cutting manual effort by 50%. LLMs performed well in extracting short-answer information and, in some cases, even outperformed human annotation. (see Section 3.6). This highlights their potential to enhance research synthesis and dataset curation in food extrusion. The approach can be extended to other domains in and out of food science requiring structured data extraction from the scientific literature.

The remainder of this paper is structured as follows. Section 2 details the dataset curation process and the role of LLMs in information extraction. Section 3 presents experimental results, including task definitions, model selection, and setup of experiments. Section 4 discusses the findings along with a detailed analysis addressing the research questions. Section 5 presents the advantages and limitations of the approach. Finally, Section 6 summarises the study, highlighting key findings, limitations, and future research directions.

## 2. Materials and Methods

### 2.1. Food Extrusion Literature Database

#### 2.1.1. Literature Search and Filtering

Food extrusion research lacks a centralised, structured dataset, making it difficult to efficiently retrieve and compare data across the literature. To address this challenge, a structured dataset was manually curated from published food extrusion research by one of the authors of this study (J.P.), who has domain expertise in food extrusion. This dataset captures categorical information related to extrusion studies rather than sample-level quantitative measurements of process conditions and product attributes, providing a searchable repository that supports deeper bibliometric analysis beyond standard academic databases.

The dataset was curated through a systematic search of the Web of Science database (https://webofscience.com (accessed on 27 March 2025)), conducted over a period of approximately three months starting from 17 October 2022. The initial search strategy aimed to capture a broad range of food extrusion studies, by retrieving studies where the keywords “extrusion”, “extruder”, and “food” appeared in the Title, Abstract, or Keywords fields (Initial search syntax = “extru*” (Topic) AND “food” (Topic)). This search returned 3828 results, consisting of 3167 research articles, 419 conference proceedings, 305 reviews, and 99 book chapters. To refine the search and focus on relevant studies, additional filtering was applied to exclude papers unrelated to food processing, such as those on polymer extrusion for food packaging, and restricting results to articles under the Food Science Technology, Nutrition Dietetics, and Agriculture Dairy Animal Science Web of Science categories (Refined search syntax = “extru*” (Topic) AND “food” (Topic) NOT “packaging” (Topic). WoS Category filter: Food Science Technology, Nutrition Dietetics, and Agriculture Dairy Animal Science). This refinement narrowed the dataset to 1910 research articles. Each article was then manually screened by the domain expert and assessed for relevance, with inclusion criteria based on whether the study contained a detailed experimental design and methodological descriptions of extrusion processing. Studies were then categorised by product type (e.g., meat analogue, snack, breakfast cereal, animal feed, pasta, rice analogue, noodle, general). To enhance dataset completeness, citation screening was conducted. This involved examining the reference lists of an extensive sample of the retrieved articles to identify additional relevant publications that were not captured in the initial or refined search. After cross-referencing and filtering to prevent article duplication, the final dataset consisted of 335 food extrusion studies. The publications span from 1997 to 2023, providing a comprehensive view of the evolving research landscape in food extrusion.

#### 2.1.2. Manual Data Extraction

A manual data extraction process was conducted to systematically capture key parameters relevant to food extrusion research. The extracted data were organised under a predefined schema, which is outlined below, ensuring consistency and comparability across studies. The dataset schema was designed to cover all aspects of extrusion processes and enable efficient information retrieval and standardisation of diverse experimental setups. The extracted data *attributes* were categorised as follows (hereafter referred to as *the schema*):

- **Publication details:** Includes metadata such as study ID within the dataset, study title, publication year, journal, journal impact factor, corresponding author country, article keywords, and the availability of quantitative data. These details provide context on the credibility and accessibility of each study and allow users to track bibliometric trends in food extrusion research.
- **Product information:** Specifies the product type(s) investigated in the study (e.g., meat analogue, breakfast cereal, snack), which is valuable for identifying application-specific trends and evaluating the data in the appropriate context, as extrusion processing conditions and characterisation methods often vary significantly depending on the type of product.
- **Process details:** Captures key details of the extruder process, including the extruder model, manufacturer, manufacturer country, extrusion screw type (i.e., single or twin screw), screw diameter (mm), length-to-diameter ratio, die dimensions (mm), and the scale of the extrusion setup (i.e., lab, pilot, or commercial scale). These parameters define the operational constraints of the extrusion process and help with comparing equipment setups across different studies.
- **Formulation details:** Describes the ingredients used within the product formulation and the in-barrel moisture content (%), both of which significantly influence the structural, textural, nutritional, and sensorial properties of extruded products. This category is important for understanding how raw material composition affects extrusion outcomes.
- **Variables and characterisation:** Categorises input variables (i.e., factors selected as independent variables in the experimental design), response variables (i.e., system dynamics of the extrusion process such as residence time and specific mechanical energy), feed characterisation metrics (i.e., characterisation performed on the raw feed ingredients), and extrudate characterisation metrics, which includes both quantitative (e.g., numerical measurements) and qualitative (e.g., image-based) methods.
- **Overall study information:** Includes a subjective rating (out of 10) by the domain expert and a paragraph summary of the study. These additional insights help contextualise studies, helping researchers to filter studies based on relevance and identify overarching themes in food extrusion research.

#### 2.1.3. Study Exclusion

After extraction, studies focusing on single-screw extrusion were excluded from the dataset, as twin-screw extrusion is widely used in food production due to its superior mixing efficiency, process control, and adaptability to complex formulations [25]. In addition, during the dataset refinement process, studies with substantial missing data were removed to improve dataset quality and reliability. However, some missing values remain in the final dataset due to incomplete data reporting in the original studies. These quality issues reflect the inherent inconsistencies in literature data reporting and extrusion experimental design rather than errors in manual data extraction. The finalised dataset consisted of 234 studies, providing a well-structured and high-quality resource for food extrusion research across a comprehensive coverage of extruded food product types.

### 2.2. LLM-Powered Information Extraction Pipeline

**Figure 1:**
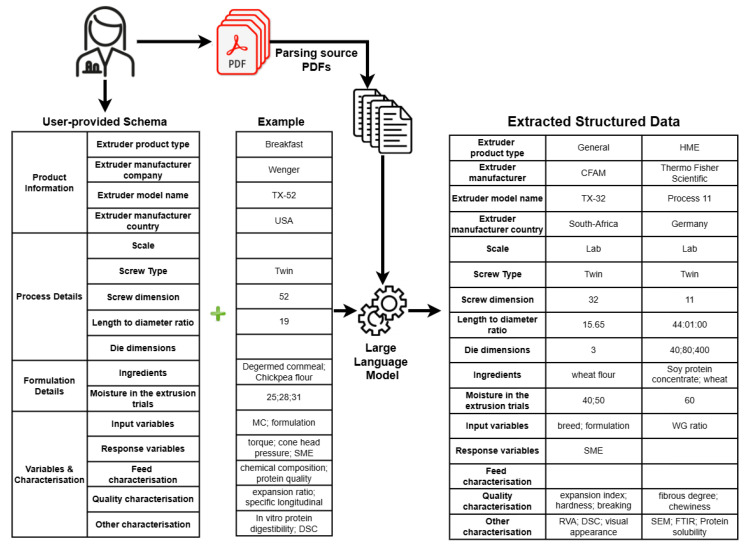
The extraction of structured data from the scientific literature utilising an existing manual-annotated dataset as examples for few-shot learning in an LLM prompt.

To automatically extract data from additional documents, we developed a pipeline that includes three main steps: (1) parsing source PDFs; (2) information extraction using LLMs; and (3) postprocessing. The pipeline is illustrated in Figure 1, which highlights the three main steps involved in processing the documents. We explain each step in detail below.

#### 2.2.1. Parsing Source PDFs

The first step of the pipeline is converting PDFs to text. The PDFs are collected by a domain expert (see Section 2.1). We use GROBID [26] (https://github.com/kermitt2/grobid (accessed on 27 March 2025)) to convert the article PDFs to XML format. The XML output contains metadata such as section names and page numbers, along with the textual content. GROBID does not process figures, and the quality of the extracted tables is poor [27]. To address this, we use an in-house table extractor to extract tables as text and their corresponding page numbers. We then concatenate the text for each section, including section names, and add the tables based on their page numbers to generate the final output.

#### 2.2.2. Information Extraction Using LLMs

To extract information, we provide the LLM with an instruction based on the schema provided by the researcher (see Section 2.1.2). The process can proceed in one of two ways:

- **Zero-shot learning:** The LLM extracts information from a set of articles without benefiting from the existing human-annotated data (the dataset in Section 2.1). The LLM is expected to generate the correct structured output based solely on the provided document and any inherent ability to understand the schema.
- **Few-shot learning:** In the few-shot setting, we use in-context learning (ICL) [9,28], which involves providing the LLM with a set of examples as part of the prompt in addition to the data to be analysed. Here, the examples are drawn from the dataset in Section 2.1. These examples help the model understand how to map schema data attributes to the desired outputs. We use 1-shot prompting following Ghosh et al. [6]. As ICL has been shown to be sensitive to the provided examples [29,30], selecting appropriate examples is crucial for improving performance. Instead of relying on random sampling, various strategies exist for selecting examples, including semantic-based methods such as KATE [15] and vocabulary-based approaches such as BM25 [31,32]. In this study, we use BM25 due to its simplicity and computational efficiency in this study. Originally developed as a bag-of-words retrieval model, *BM25* [33] scores to retrieve articles from the human-corrected dataset (training examples) by measuring the textual similarity to the article being analysed. By selecting contextually similar articles, the intuition is that the examples of extracted data will be more relevant to the article at hand, thereby improving the LLM’s performance in extracting information.

#### 2.2.3. Postprocessing

One of the challenges of LLMs is the postprocessing of the LLM-generated answer. Therefore, we follow Ghosh et al. [6] and use JSON format as the output format of the instruction. We use Python’s built-in json library (https://docs.python.org/3/library/json.html (accessed on 27 March 2025)) with recursive parsing supporting objects, arrays, strings, and numbers while also managing edge cases, such as missing values within objects.

#### 2.2.4. Large Language Models

Pre-trained language models (PLMs) and LLMs have demonstrated remarkable performance in AI applications; however, LLMs offer significant advantages over PLMs in many use cases [34,35]. While PLMs are effective for specific tasks, they typically require fine-tuning to adapt to specialised domains or tasks [36,37]. Fine-tuning is resource-intensive, limiting the flexibility and scalability of PLMs in real-world applications. Moreover, PLMs have a limited context window size, which restricts their performance when handling large volumes of text, such as full-text scientific articles, where the model needs to grasp long-range dependencies and contextual information spread across several pages. As a result, we choose to use LLMs in the study.

We use LLMs from two different categories: (1) proprietary LLMs, including GPT4 (GPT-4o, o1-mini) [11], Claude (Claude v1, Claude 3 Sonnet) [12], and Gemini (Gemini 1.5 Pro) [13,38]; and (2) open-source LLMs, including Llama (Llama-3.1-8B-Instruct, Llama-3.1-70B-Instruct, and Llama-3.1-405B-Instruct) [39], Mistral (Mistral-7B-Instruct-v0.2) [40], and Nemotron (Llama3.1-70B Nemotron) [41]. We choose LLMs based on their context length, which is important as we provide full-text scientific articles as input in the instruction. These articles range in length from 6 to 29 pages.

A comparison of the LLMs in terms of context length, response time, and cost is presented in Table 1. In addition to context length, we also evaluate latency (response time) and cost. For proprietary LLMs, latency is measured as the time between sending the API request and receiving the first token. For open-source LLMs, it measures the time taken to generate the completion. At the time of writing, the cost (last accessed on 19 December 2024), expressed in USD per million tokens, is calculated using a weighted average of input and output token prices (3:1 ratio).

## 3. Results

### 3.1. Dataset

For the experiments, we split the human-annotated dataset (see Section 2.1) into 150 test examples and 84 training examples. As we are using the in-context learning variant of few-shot learning, the training examples are used as a selection pool to find a prior example that can guide the LLM inference (see Section 2.2.2). As such, due to the nature of this specific “training”, we do not require the standard data splits for training, development, and validation. Instead, we used a larger test set to ensure a more reliable evaluation of the method, given that the human-annotated dataset is small (only 234 articles). The training examples are used for few-shot learning, as we described in Section 2.2.

### 3.2. Evaluation Metrics

To assess the performance of extracting structured data from articles, we employ three standard evaluation metrics: precision, recall, and F_1_ score, following the approach of Ghosh et al. [6].

**Precision** evaluates the accuracy of the extracted data by calculating the proportion of retrieved data correctly identified (true positives) out of all data marked as retrieved by the model (true positives + false positives).

(1) $$\mathrm{Precision}=\frac{\mathrm{True}\;\mathrm{Positives}}{\mathrm{True}\;\mathrm{Positives}+\mathrm{False}\;\mathrm{Positives}}$$

**Recall** measures the model’s capacity to capture all relevant data by calculating the proportion of correctly extracted retrieved data (true positives) out of all actual retrieved data in the dataset (true positives + false negatives).

(2) $$\mathrm{Recall}=\frac{\mathrm{True}\;\mathrm{Positives}}{\mathrm{True}\;\mathrm{Positives}+\mathrm{False}\;\mathrm{Negatives}}$$

**F_1_ score** is the harmonic mean of precision and recall.

(3) $$F_{1}\;\mathrm{Score}=2\times \frac{\mathrm{Precision}\times \mathrm{Recall}}{\mathrm{Precision}+\mathrm{Recall}}$$

### 3.3. Automatic Evaluation

To understand the impact of examples in the pipeline, we ran the experiments on the test set using zero-shot and 1-shot (few-shot) settings. We report the results in Table 2 for both settings. From the results, it is evident that providing example outputs (1-shot) significantly improves the performance of all models, as it helps clarify the expected output format for a given schema (e.g., extruder manufacturer country: *USA*, length to diameter ratio: *5;20;200*). This approach helps the model distinguish between relevant and irrelevant details, leading to more accurate extractions (e.g., for die dimensions: **zero-shot:** *slit viscometer die (2 × 20 × 100 mm)*, **1-shot:** *2;20;100*, **ground-truth:** *2;20;100*). For the zero-shot and the 1-shot settings, the best model was the Llama 3.1 405B model, an open-source model. Interestingly, the Llama 3.1 70B model was competitive with the best proprietary model (GPT-4o), highlighting that smaller open-source models are viable solutions here, particularly given their low cost.

Our results show that open-source models typically perform better than proprietary models at a much lower cost (see Table 1). This highlights the importance of research groups being able to use local open-source models without performance degradation. These models can be deployed locally, which ensures data privacy. Additionally, open-source models promote fairness and collaboration through reproducibility and open benchmarking, making them a good alternative to proprietary models, especially in few-shot (1-shot) learning where examples are used.

### 3.4. Impact of Training Set Size

To understand the effect of dataset size on the 1-shot (few-shot) learning method, we conduct experiments with different training set sizes, varying the number of ground truth examples (the large set contains all examples in the train set). As noted, we use the examples to select the most similar article by BM25 (see Section 2.2.2). We also use three different randomly sampled sets of articles for the small and medium sizes to observe the effects of variance across samples. Our results are shown for the Llama 3.1 70B model as this represents an attractive model that balances cost (see Table 1) and performance (see Table 2). The results, along with the zero-shot results, are shown in Table 3. In particular, for small sets, the selected articles (and their outputs) significantly impact the predictions. Finally, the performance difference between the medium and large sets is minimal, suggesting that increasing the set size beyond a certain point does not yield substantial improvements.

### 3.5. Results per Data Attribute Class

In Table 4, we give the performance results per data attribute class for zero-shot and 1-shot settings. Here, we report results using the Claude v1 LLM to examine the quality issues with the worse-performing LLM, noting that quality will be improved with any other tested LLM. The table shows that 1-shot significantly outperforms zero-shot across all data attributes for the provided schema, highlighting the value of few-shot learning in improving model accuracy. The 1-shot results show notable improvements in data attributes like extruder product type and screw type, in which provided examples relate to typological distinctions in the science domain. However, data attributes, such as other characterisation and feed characterisation, are article-specific and involve longer form information with a reduction in content overlap, highlighting challenges in comparing and evaluating AI-generated extractions and the human-extracted information (e.g., for other characterisation: **1-shot:** *XRD, micro-CT, FT-IR, DSC, RVA, in vitro digestion model*, **ground-truth:** *visual appearance; XRD; pasting properties; DSC; FTIR; in vitro digestion*).

### 3.6. Additional Content Verification Annotations

As highlighted above, the extract string comparison metrics of precision, recall, and F_1_ are overly strict and do not cater well to situations where the AI-generated results and the human-authored data differ only in a trivial manner without negatively affecting the *content* extracted. To investigate this issue, we conducted an additional annotation task to assess these differences.

The annotation was performed on 20 articles, allowing us to examine the impact of the provided examples on extraction accuracy. The domain expert (J.P.), who also curated the manually extracted dataset, assessed the extracted information based on its adherence to the predefined schema. The extracted data were categorised into four groups: (1) Better than human; (2) Correct; (3) Partially correct (that is, correct but missing some details); and (4) Wrong.

**Figure 2:**
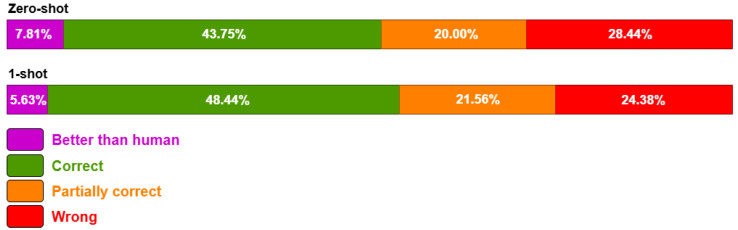
Content verification annotations for the quality of the structured table across four categories.

The distribution of results across the human annotation categories is presented in Figure 2, where the proportion of each category is shown as a bar with corresponding percentages. We compare F_1_ scores with the sum of *Better than human* and *Correct* because these categories represent fully acceptable extractions. The discrepancy between results highlights the importance of human validation, as automatic metrics may overlook context-dependent correctness. Unlike F_1_, which strictly measures exact matches, human validation considers partial correctness and contextual validity, leading to higher accuracy percentages and better capturing of nuanced extractions. When we aggregated the results from the *Better than human* and *Correct* categories, we found that 51.56% and 54.07% of the 360 cells (16 columns × 20 articles) were accurate in the zero-shot and 1-shot settings, respectively. With this analysis, we treat the exact match results as a *lower bound* on performance, noting that the actual utility may be much higher than suggested by the absolute values of precision, recall, and F_1_ (note that the F_1_ scores for the zero-shot and 1-shot settings were 10.59 and 29.86, respectively).

The domain expert also stated several key observations. Both zero-shot and 1-shot settings produce similar accuracy, noting that even the zero-shot setting outperformed human experts in providing accurate information in some cases. The 1-shot setting achieved slightly higher accuracy (human annotation) and resulted in fewer incorrect extractions compared to the zero-shot setting. Both zero-shot and 1-shot settings struggled with extracting information for extruder product type and response variables. Interestingly, in the 1-shot setting, the model frequently included input variables that are held constant in the study. Ideally, input variables should only include factors that change during the experiment, yet the model often misclassified fixed study parameters as variables. The *Correct* and *Better than human* categories are concentrated in data attributes with short-answer responses, such as scale, extruder manufacturer country, and extruder model name, in both settings, suggesting that LLMs perform best on structured, well-defined fields with limited possible responses. Some model-generated answers are correct but not identical to the ground truth; for example, the ground truth may list “Twin”, while the model extracted “co-rotating twin-screw”, which is a more specific but still valid response.

**Figure 3:**
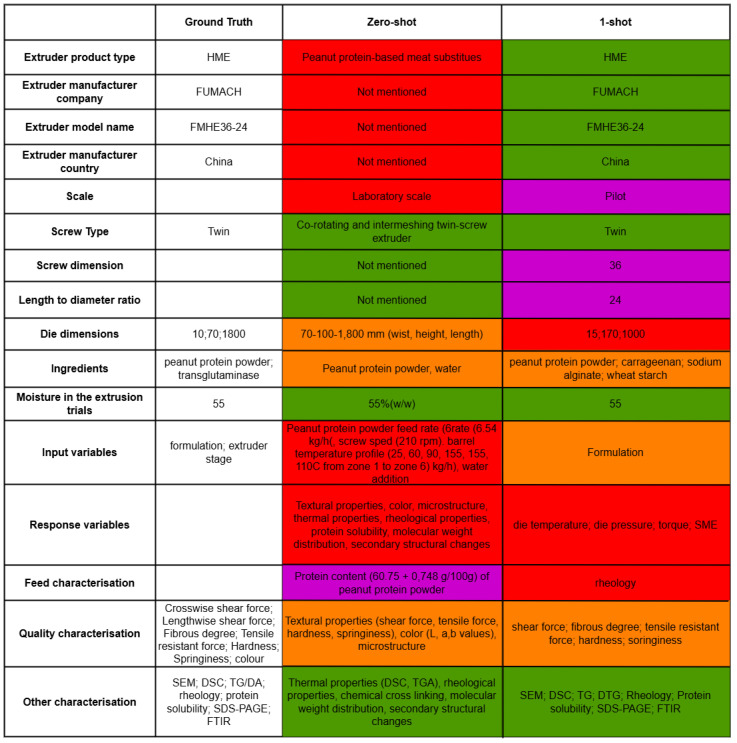
An example article with ground truth and extracted data in zero-shot and 1-shot settings, along with content verification annotation categories (see Section 3.6).

An example article with ground truth and extracted data in zero-shot and 1-shot settings is given in Figure 3 with the categories the domain expert used for content verification annotation. We selected the example that includes all categories that are domain-expert defined. The figure visually demonstrates the differences between the ground truth and extracted information with zero-shot and 1-shot.

## 4. Discussion

### 4.1. Outcomes and Challenges

We introduce a manually constructed dataset for food extrusion and show how it could be used to automate the creation of supplementary data. Our use of LLMs benefits from the manually constructed dataset through the few-shot learning technique. Our results show that open-source models are competitive with proprietary models and that the few-shot method provides greater performance than the zero-shot method, even when there are limited data available. Furthermore, we show that the percentage of high-quality data is high; approximately 75% of the generated data were classed as correct when validated by a human expert (see Section 3.6). While this shows that LLMs are useful and capable of generating high-quality data, a human process is required to identify incorrect data. We note that this validation and correction activity is an easier task than extracting the data from scratch.

### 4.2. LLM-Powered Literature Review

When compared to manual human annotation, LLMs are significantly faster and can process large quantities of data in a short amount of time. While human annotators require substantial time to read and interpret each paper, LLMs can extract relevant data in seconds. However, humans have the advantage of domain-specific expertise, which helps them interpret complex relationships and identify data points that may be context-dependent or non-standardised in the literature. Studies have explored using LLMs as annotators for various NLP tasks [42,43,44,45]. Fully automated data annotation would free human annotators and reduce annotation costs; however, studies show that the performance of LLMs depends on tasks, datasets, and labels [46,47]. This highlights the continued need for human expertise, especially in tasks that require domain-specific knowledge. While LLMs can help speed up the process and reduce effort, human validation is essential to ensure accurate and reliable results. Combining the strengths of both can lead to better, more reliable annotations. Therefore, we argue for a pipeline that extracts information according to a schema provided by a human expert, keeping the human expert involved in the process to provide examples and improve the accuracy of the system.

In our study, we found that while LLMs performed well in automating the extraction process, their results were more accurate when combined with human-provided examples (few-shot learning). It shows that human expertise plays a crucial role in overseeing the LLM’s output. Experts can ensure that the data extracted by LLMs is contextually accurate and aligned with current industry standards. Human involvement can also help resolve ambiguities in the literature, which LLMs may struggle with, and provide the necessary domain-specific knowledge to refine the model’s annotations. This hybrid approach, where LLMs are used to accelerate the annotation process and humans provide oversight and domain-specific validation, offers the best of both worlds: efficiency and accuracy.

### 4.3. Cost and Energy Consumption of LLM-Powered IE

We use NVIDIA H100 GPUs for inference of open-source LLMs. Our experiments are conducted on a node with two × NVIDIA H100 GPUs, and we track energy consumption using CodeCarbon [48]. For a 1-minute run, the estimated carbon emissions are approximately 0.0228 grams of CO_2_. The average cost per model for a single call per article in the test is given in Table 1. The total cost for each model can be calculated as the cost per call multiplied by the number of articles.

When we compare the performance versus cost and latency of models, we find that the Llama 3.1 405B model is the best overall for its strong accuracy, especially in the 1-shot setting. While it is more expensive and slower, its performance makes it ideal for tasks that need high accuracy. If cost and faster responses are more important, models like Llama 3.1 8B offer a good balance of lower cost, speed, and decent performance. Among proprietary and open-source LLMS, GPT-4 and Claude offer high performance but come with high costs and less flexibility. They are easy to use and are well supported but may have limitations in customisation and usage restrictions. On the other hand, open-source LLMs are more cost-effective and customisable. They allow more control and privacy but require technical expertise to set up.

### 4.4. Automatic Evaluation of LLM-Powered IE

We use precision, recall, and F_1_ scores based on the exact match as evaluation metrics of the system. Exact match metrics ignore the syntactic differences between generated and ground truth responses, penalising models for generating valid but non-identical responses. However, from the additional round of human annotations, we find that even though the extracted information does not directly match with ground truth, they are correct or even better than them (see Section 3.6). We conclude that exact-match metrics are not ideal for evaluating LLM predictions, as they fail to account for the different ways a correct answer can be phrased. Instead, more effective metrics, such as METEOR [49] and SARI [50], which are text generation evaluation metrics that account for paraphrasing and synonymy, can be considered. Specifically, METEOR improves upon exact match by incorporating synonyms, stemming, and paraphrasing to better capture meaning and linguistic variations while also considering word order. SARI measures the percentage of correctly added, kept, and deleted n-grams across input, output, and reference sentences, making it particularly useful for assessing content transformation. These metrics consider meaning and word choice, offering a more nuanced evaluation of the model’s ability to generate relevant and accurate content. Additionally, we can also further employ human judgements to provide a better assessment of semantic similarity.

## 5. Advantages and Limitations

### 5.1. Advantages

The proposed method offers several notable advantages in the context of scientific literature review and data extraction. It significantly reduces the time and manual effort typically required to extract relevant information from large volumes of publications. The method is scalable, allowing researchers to continuously expand their datasets with minimal additional effort. The integration of domain expertise in the few-shot learning setting of the LLM enhances both the accuracy and relevance of the extracted data, ensuring that the information is reliable for subsequent analysis. The approach provides a cost-effective solution by minimising the need for extensive manual data curation. Lastly, the method is flexible and can be adapted to other domains beyond food extrusion, offering a valuable tool for extracting structured data from the scientific literature across a wide range of research areas.

### 5.2. Limitations

**Dynamic update of dataset:** The proposed method for data creation can assist researchers in processing large volumes of publications. However, it relies on publications gathered by domain experts and does not include a dynamic update mechanism to integrate newly released publications unless the researcher manually adds them. As a result, the method heavily depends on periodic manual reviews by human experts to identify and add relevant papers, making it difficult to maintain up-to-date content. In the future, we will focus on automating the process of paper discovery based on relevance to the topic. This extension will help streamline dataset expansion and enhance its ability to stay aligned with the latest scientific developments in the field.

**Missing extracted information:** Despite the advancements in using LLMs for data extraction, they still miss critical information in certain cases. To explore the missing information that could not be extracted by the LLM, we calculate the empty cells for each column given in the schema compared with the ground truth set (including both empty cells and cells with “Not mentioned”). We find that using an example (1-shot) helps improve the extraction process, as it guides the LLM on what information to focus on in the provided article. However, despite this improvement, missing information is still a limitation.

**PDF parsing:** To extract content from PDFs, we use GROBID to convert article PDFs to XML format and an off-the-shelf table extractor for extracting tables. We found that GROBID may fail to extract information from certain PDFs. In our approach, two papers—published in 2004 and 2022—had XML parsing errors where GROBID could not extract information. These errors might be due to the complexity of the documents or specific formatting in those PDFs, which may not be fully compatible with GROBID’s parsing capabilities.

We also evaluated the table extraction performance of the table extractor. Some tables were either extracted incorrectly or were missing altogether. However, as the attributes defined by the human expert do not rely on the tables for this dataset, this did not affect the overall quality of our approach. For cases where extraction from complex tables (e.g., spanning multiple pages) is critical, the performance of the approach may degrade.

**Hallucination:** A notable issue observed is that LLM with the few-shot learning setting occasionally extracts hallucinated information that is not present anywhere in the paper. For example, in Study 19, the model extracted “flaxseed protein concentrate”, despite no mention of this ingredient in the original article. This indicates the need for a checker to control the extracted information from the given article, which will be addressed in future work.

**n-ary relation validation:** This approach extracts the information together for each paper, similar to the approach described by Ghosh et al. [6]. This approach helps capture dependencies between related fields (e.g., “Screw type” and “Screw dimension”), which we refer to as an *n*-ary relation, where *n* is the number of arguments to a relation. Currently, our method lacks a validation mechanism to verify the accuracy of these interdependencies, and this remains a limitation of this work. The validation of *n*-ary relations, which would ensure the consistency and correctness of complex relationships between extracted data, is left for future research.

## 6. Conclusions

**Merits:** We introduced a human-annotated dataset for food extrusion research and a method to supplement the human-annotated dataset, overcoming data coverage limitations inherent in the time-intensive nature of manually reviewing and structuring information in the scientific literature. The dataset captures critical parameters relevant to food extrusion studies and provides a foundation for contextualising future quantitative data extraction and predictive modelling activities. We demonstrated the capacity of LLMs to process large volumes of unstructured scientific text, extracting relevant parameters according to a predefined schema. Our experiments showed that LLMs can effectively identify key details related to extrusion conditions, equipment setups, and product characterisation methods. We explored a few-shot learning LLM approach that integrates domain expertise to better instruct LLM inference, improving both accuracy and reliability, thereby providing a method to generate supplementary data. Notably, LLMs significantly reduce the time and effort required for literature reviews. In certain instances, LLMs even outperformed human annotation, showcasing their effectiveness in automating short-answer information extraction.

**Limitations:** Despite these advantages, challenges persist, particularly in handling implicit contextual information and addressing issues such as hallucination and missing extracted information. Hallucination, where the model generates information not supported by the source text, remains a significant challenge. Additionally, some relevant details might be missed or inaccurately extracted, especially when dealing with complex or ambiguous contexts. While LLMs are effective in extracting structured data, these limitations highlight the need for continued refinement and validation, especially in highly specialised domains like food extrusion.

**Future directions:** Moving forward, the generated dataset can be leveraged to develop a domain-specific ontology and build a knowledge graph of the food extrusion domain, mapping relationships between processing conditions, material properties, and product characteristics. This structured representation could enhance research exploration, hypothesis generation, and systematic literature analysis.

A key next step could be using the dataset to fine-tune an in-domain LLM specifically tailored to food extrusion research. By fine-tuning a localised model, we would expect to improve structured data extraction accuracy, minimising errors commonly associated with general-purpose language models. These advancements have the potential to transform how the food extrusion literature is accessed, structured, and utilised, bridging the gap between manual curation and automated knowledge discovery.

These advancements have the potential to enhance literature review, reduce manual curation efforts, and accelerate research in food extrusion and beyond. As AI-driven tools evolve, they offer an opportunity to bridge the gap between raw scientific data and actionable insights, ultimately advancing data-driven decision-making in food process experimentation, design, and modelling.

## Figures and Tables

**Table 1:** Comparison of LLMs for context length, response time, and cost of usage ^1^.

Category	LLM	Context Length	Response Time (s)	Cost (USD/M Tokens)
Proprietary	GPT-4o	128k	0.40	4.38
	o1-mini	128k	14.29	5.25
	Claude v1	200k	0.78	6.00
	Claude 3 Sonnet	200k	0.78	6.00
	Gemini 1.5 Pro	2m	0.74	2.19
Open-source	Llama 3.1 8B	128k	0.37	0.11
	Llama 3.1 70B	128k	0.43	0.84
	Llama 3.1 405B	128k	0.69	6.25
	Mistral-7B	8k	0.28	0.18
	Llama 3.1 Nemotron 70B	128k	0.56	0.27

^1^ https://artificialanalysis.ai/leaderboards/models (accessed on 27 March 2025).

**Table 2:** Results of extracting structured data from articles. Based on the F_1_ score, the best results are **boldfaced** and the second-best is underlined for zero-shot and 1-shot settings.

Model	Zero-Shot			1-Shot		
	Precision	Recall	F_1_ Score	Precision	Recall	F_1_ Score
GPT-4o	14.47	14.26	14.36	29.30	28.46	28.87
o1-mini	14.38	14.00	14.19	19.76	18.75	19.24
Claude 3 Sonnet	11.18	10.96	11.07	26.59	26.06	26.33
Claude v1	11.15	10.08	10.59	30.42	29.32	29.86
Llama 3.1 8B	12.66	12.33	12.49	30.55	29.62	30.08
Llama 3.1 70B	14.27	14.24	14.26	30.46	29.76	30.11
Llama 3.1 405B	14.45	14.32	**14.38**	31.17	30.11	**30.64**
Mistral-7B	10.48	10.10	10.19	28.17	27.12	27.23
Nemotron 70B	13.75	13.14	13.24	29.52	28.45	28.78

**Table 3:** Results of extracting structured data based on different sizes of the training set. The best results are **boldfaced**, and the second-best is underlined.

Size	Precision	Recall	F_1_ Score
Zero-shot	14.27	14.24	14.26
Small (10 articles)	25.12	23.56	24.34
	19.57	18.56	19.06
	19.61	19.71	19.66
Medium (50 articles)	30.84	29.31	30.08
	31.11	29.88	29.99
	29.73	27.56	28.65
Large (84 articles)	30.46	29.76	**30.11**

**Table 4:** Results per data attribute class. Based on the F_1_ score, the best results are **boldfaced** for each data attribute.

Schema	Zero-Shot			1-Shot		
	Precision	Recall	F_1_	Precision	Recall	F_1_
Extruder product type	2.78	2.67	2.72	52.35	52.35	**52.35**
Extruder manufacturer company	36.51	30.67	33.33	49.65	46.98	**48.28**
Extruder model name	41.67	36.67	39.01	55.00	51.68	**53.29**
Extruder manufacturer country	74.60	62.67	68.12	77.70	72.48	**75.00**
Scale	1.33	1.33	1.33	21.48	21.48	**21.48**
Screw type	0.00	0.00	0.00	91.72	89.26	**90.47**
Screw dimension	0.00	0.00	0.00	24.24	21.48	**22.78**
Length to diameter ratio	31.25	20.00	24.39	24.60	20.81	**22.55**
Die dimensions	0.00	0.00	0.00	27.86	26.17	**26.99**
Ingredients	6.67	6.67	6.67	16.78	16.78	**16.78**
Moisture in the extrusion trials	0.00	0.00	0.00	29.25	28.86	**29.05**
Input variables	0.00	0.00	0.00	12.75	12.75	**12.75**
Response variables	0.00	0.00	0.00	4.03	4.03	**4.03**
Feed characterisation	0.00	0.00	0.00	0.69	0.67	**0.68**
Quality characterisation	0.00	0.00	0.00	0.67	0.67	**0.67**
Other characterisation	0.00	0.00	0.00	0.00	0.00	0.00

## Data Availability

Pennells, Jordan (2025): A Structured Human-Annotated Dataset for Food Extrusion Literature. v1. CSIRO. Data Collection. https://doi.org/10.25919/r4y6-r260.

## References

[1] Harper J.M., Clark J.P. (1979). Food extrusion. Crit. Rev. Food Sci. Nutr..

[2] Singh S., Gamlath S., Wakeling L. (2007). Nutritional aspects of food extrusion: A review. Int. J. Food Sci. Technol..

[3] Choton S., Gupta N., Bandral J.D., Anjum N., Choudary A. (2020). Extrusion technology and its application in food processing: A review. Pharma Innov. J..

[4] Dalbhagat C.G., Mahato D.K., Mishra H.N. (2019). Effect of extrusion processing on physicochemical, functional and nutritional characteristics of rice and rice-based products: A review. Trends Food Sci. Technol..

[5] Borg C.K., Frey C., Moh J., Pollock T.M., Gorsse S., Miracle D.B., Senkov O.N., Meredig B., Saal J.E. (2020). Expanded dataset of mechanical properties and observed phases of multi-principal element alloys. Sci. Data.

[6] Ghosh S., Brodnik N., Frey C., Holgate C., Pollock T., Daly S., Carton S. Toward Reliable Ad-hoc Scientific Information Extraction: A Case Study on Two Materials Dataset. Proceedings of the Findings of the Association for Computational Linguistics: ACL 2024.

[7] Fukagawa N.K., McKillop K., Pehrsson P.R., Moshfegh A., Harnly J., Finley J. (2022). USDA’s FoodData Central: What is it and why is it needed today?. Am. J. Clin. Nutr..

[8] Caracciolo C., Stellato A., Morshed A., Johannsen G., Rajbhandari S., Jaques Y., Keizer J. (2013). The AGROVOC linked dataset. Semant. Web.

[9] Brown T.B. (2020). Language models are few-shot learners. arXiv.

[10] Touvron H., Martin L., Stone K., Albert P., Almahairi A., Babaei Y., Bashlykov N., Batra S., Bhargava P., Bhosale S. (2023). Llama 2: Open foundation and fine-tuned chat models. arXiv.

[11] Achiam J., Adler S., Agarwal S., Ahmad L., Akkaya I., Aleman F.L., Almeida D., Altenschmidt J., Altman S., Anadkat S. (2023). Gpt-4 technical report. arXiv.

[12] Anthropic (2024). The Claude 3 Model Family: Opus, Sonnet, Haiku. https://www.anthropic.com/news/claude-3-family.

[13] Team G., Anil R., Borgeaud S., Alayrac J.B., Yu J., Soricut R., Schalkwyk J., Dai A.M., Hauth A., Millican K. (2023). Gemini: A family of highly capable multimodal models. arXiv.

[14] Wei J., Wang X., Schuurmans D., Bosma M., Chi E., Le Q., Zhou D. (2022). Chain of thought prompting elicits reasoning in large language models. arXiv.

[15] Liu J., Shen D., Zhang Y., Dolan W.B., Carin L., Chen W. What Makes Good In-Context Examples for GPT-3?. Proceedings of the Deep Learning Inside Out (DeeLIO 2022): The 3rd Workshop on Knowledge Extraction and Integration for Deep Learning Architectures.

[16] Hegselmann S., Buendia A., Lang H., Agrawal M., Jiang X., Sontag D. Tabllm: Few-shot classification of tabular data with large language models. Proceedings of the International Conference on Artificial Intelligence and Statistics.

[17] Morris M.R. (2023). Scientists’ Perspectives on the Potential for Generative AI in their Fields. arXiv.

[18] Hope T., Downey D., Weld D.S., Etzioni O., Horvitz E. (2023). A computational inflection for scientific discovery. Commun. ACM.

[19] Bolanos F., Salatino A., Osborne F., Motta E. (2024). Artificial intelligence for literature reviews: Opportunities and challenges. Artif. Intell. Rev..

[20] Olivetti E.A., Cole J.M., Kim E., Kononova O., Ceder G., Han T.Y.J., Hiszpanski A.M. (2020). Data-driven materials research enabled by natural language processing and information extraction. Appl. Phys. Rev..

[21] Şahinuç F., Tran T., Grishina Y., Hou Y., Chen B., Gurevych I. Efficient Performance Tracking: Leveraging Large Language Models for Automated Construction of Scientific Leaderboards. Proceedings of the 2024 Conference on Empirical Methods in Natural Language Processing.

[22] Peng R., Liu K., Yang P., Yuan Z., Li S. (2023). Embedding-based retrieval with llm for effective agriculture information extracting from unstructured data. arXiv.

[23] Zheng Z., Zhang O., Borgs C., Chayes J.T., Yaghi O.M. (2023). ChatGPT chemistry assistant for text mining and the prediction of MOF synthesis. J. Am. Chem. Soc..

[24] Wan S., Bölücü N., Duenser A., Irons J., Lee C., Jin B., Rybinski M., Walker S., Yang H. (2025). A Case for User-Centred NLP Methodology: Developing a Science Literature AI Assistant CSIRO Technical Report. Id:EP2025-1250. CSIRO. https://publications.csiro.au/publications/publication/PIcsiro:EP2025-1250.

[25] Bouvier J. (2010). Twin screw versus single screw in feed extrusion processing. Proceedings of the Extrusion Technology in Feed and Food Processing.

[26] Lopez P. (2009). GROBID: Combining automatic bibliographic data recognition and term extraction for scholarship publications. Proceedings of the Research and Advanced Technology for Digital Libraries: 13th European Conference, ECDL 2009.

[27] Meuschke N., Jagdale A., Spinde T., Mitrović J., Gipp B. (2023). A benchmark of pdf information extraction tools using a multi-task and multi-domain evaluation framework for academic documents. Proceedings of the International Conference on Information.

[28] Radford A., Wu J., Child R., Luan D., Amodei D., Sutskever I. (2019). Language models are unsupervised multitask learners. OpenAI Blog.

[29] Lu Y., Bartolo M., Moore A., Riedel S., Stenetorp P. Fantastically Ordered Prompts and Where to Find Them: Overcoming Few-Shot Prompt Order Sensitivity. Proceedings of the 60th Annual Meeting of the Association for Computational Linguistics (Volume 1: Long Papers).

[30] Agrawal S., Zhou C., Lewis M., Zettlemoyer L., Ghazvininejad M. In-context Examples Selection for Machine Translation. Proceedings of the Findings of the Association for Computational Linguistics: ACL 2023.

[31] Xu Y., Zhu C., Wang S., Sun S., Cheng H., Liu X., Gao J., He P., Zeng M., Huang X. (2021). Human parity on commonsenseqa: Augmenting self-attention with external attention. arXiv.

[32] Wang S., Xu Y., Fang Y., Liu Y., Sun S., Xu R., Zhu C., Zeng M. Training Data is More Valuable than You Think: A Simple and Effective Method by Retrieving from Training Data. Proceedings of the 60th Annual Meeting of the Association for Computational Linguistics (Volume 1: Long Papers).

[33] Robertson S., Zaragoza H. (2009). The probabilistic relevance framework: BM25 and beyond. Found. Trends® Inf. Retr..

[34] Xu D., Chen W., Peng W., Zhang C., Xu T., Zhao X., Wu X., Zheng Y., Wang Y., Chen E. (2024). Large language models for generative information extraction: A survey. Front. Comput. Sci..

[35] He K., Mao R., Lin Q., Ruan Y., Lan X., Feng M., Cambria E. (2025). A survey of large language models for healthcare: From data, technology, and applications to accountability and ethics. Inf. Fusion.

[36] Gutiérrez B.J., McNeal N., Washington C., Chen Y., Li L., Sun H., Su Y. Thinking about GPT-3 In-Context Learning for Biomedical IE? Think Again. Proceedings of the Findings of the Association for Computational Linguistics: EMNLP 2022.

[37] Bölücü N., Rybinski M., Wan S. Impact of sample selection on in-context learning for entity extraction from scientific writing. Proceedings of the Findings of the Association for Computational Linguistics: EMNLP 2023.

[38] Team G.G. (2024). Gemini 1.5: Unlocking Multimodal Understanding Across Millions of Tokens of Context. https://goo.gle/GeminiV1-5.

[39] Meta (2024). Llama3 Model Card. https://huggingface.co/collections/meta-llama/.

[40] Jiang A.Q., Sablayrolles A., Mensch A., Bamford C., Chaplot D.S., de las Casas D., Bressand F., Lengyel G., Lample G., Saulnier L. (2023). Mistral 7B. arXiv.

[41] NVIDIA (2024). Llama-3_1-Nemotron-70B-Instruct | NVIDIA NIM. https://build.nvidia.com/nvidia/llama-3_1-nemotron-70b-instruct/modelcard.

[42] Gilardi F., Alizadeh M., Kubli M. (2023). ChatGPT outperforms crowd workers for text-annotation tasks. Proc. Natl. Acad. Sci. USA.

[43] He X., Lin Z., Gong Y., Zhang H., Lin C., Jiao J., Yiu S.M., Duan N., Chen W. (2023). Annollm: Making large language models to be better crowdsourced annotators. arXiv.

[44] Aldeen M., Luo J., Lian A., Zheng V., Hong A., Yetukuri P., Cheng L. ChatGPT vs. Human Annotators: A Comprehensive Analysis of ChatGPT for Text Annotation. Proceedings of the 2023 International Conference on Machine Learning and Applications (ICMLA).

[45] Törnberg P. (2023). Chatgpt-4 outperforms experts and crowd workers in annotating political twitter messages with zero-shot learning. arXiv.

[46] Zhu Y., Zhang P., Haq E.U., Hui P., Tyson G. (2023). Can chatgpt reproduce human-generated labels? a study of social computing tasks. arXiv.

[47] Ziems C., Held W., Shaikh O., Chen J., Zhang Z., Yang D. (2024). Can large language models transform computational social science?. Comput. Linguist..

[48] Schmidt V., Goyal K., Joshi A., Feld B., Conell L., Laskaris N., Blank D., Wilson J., Friedler S., Luccioni S. CodeCarbon: Estimate and Track Carbon Emissions from Machine Learning Computing. 2021, 4658424. https://zenodo.org/records/11171501.

[49] Banerjee S., Lavie A. METEOR: An Automatic Metric for MT Evaluation with Improved Correlation with Human Judgments. Proceedings of the ACL Workshop on Intrinsic and Extrinsic Evaluation Measures for Machine Translation and/or Summarization.

[50] Xu W., Napoles C., Pavlick E., Chen Q., Callison-Burch C. (2016). Optimizing statistical machine translation for text simplification. Trans. Assoc. Comput. Linguist..

